# Supplementary figures and images for: A pH-sensitive switch activates virulence in Salmonella
Source: eLife. 2023 Sep 14;12:e85690. doi: 10.7554/eLife.85690 (PMC10519707; doi:10.7554/eLife.85690)

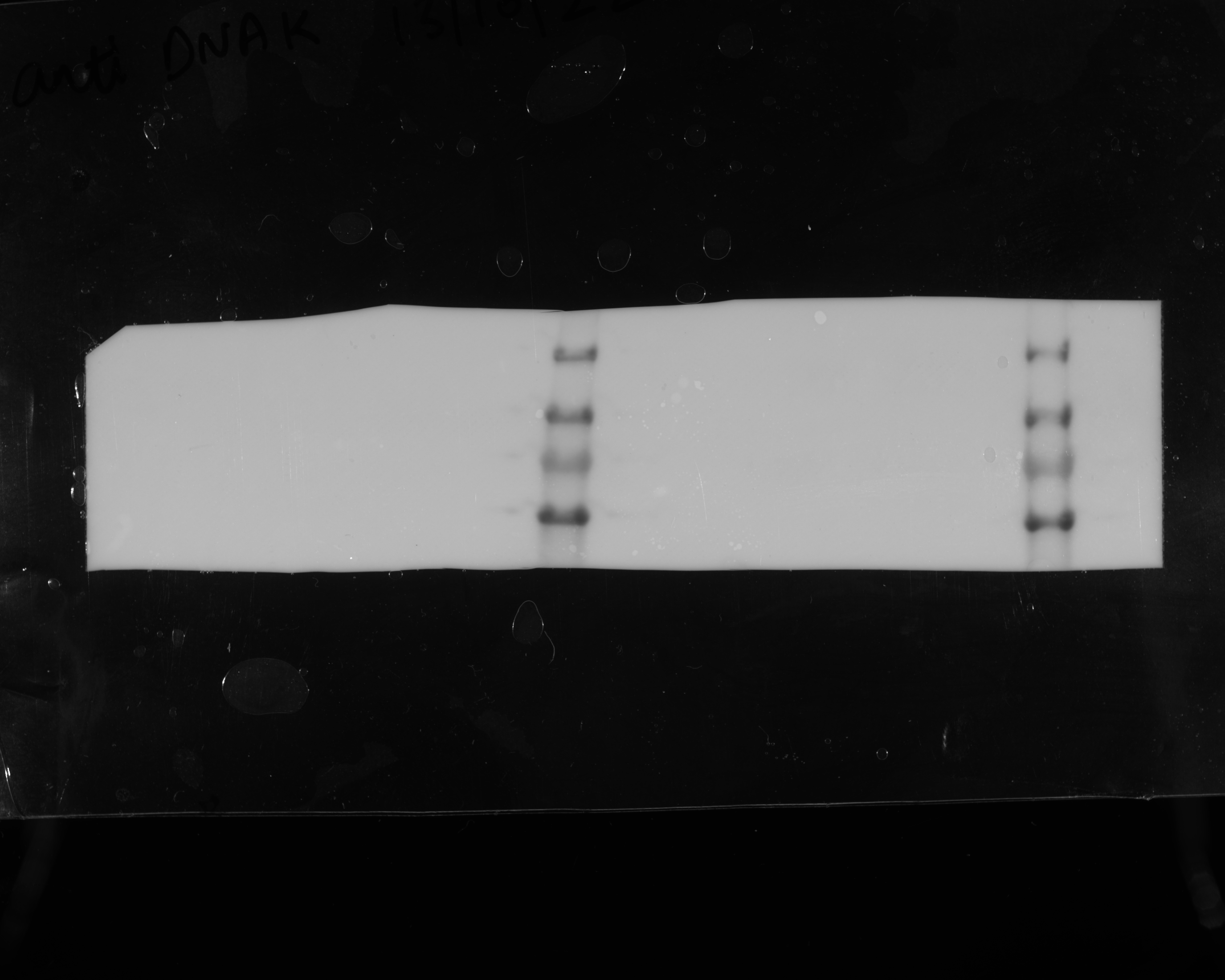

Supplement: Figure 3—figure supplement 3—source data 1. [file elife-85690-fig3-figsupp3-data1.zip › Figure 3- figure supplement 3- source data 1/anti-DNAK-oct13_2022_2(Colorimetric).raw16.tif]

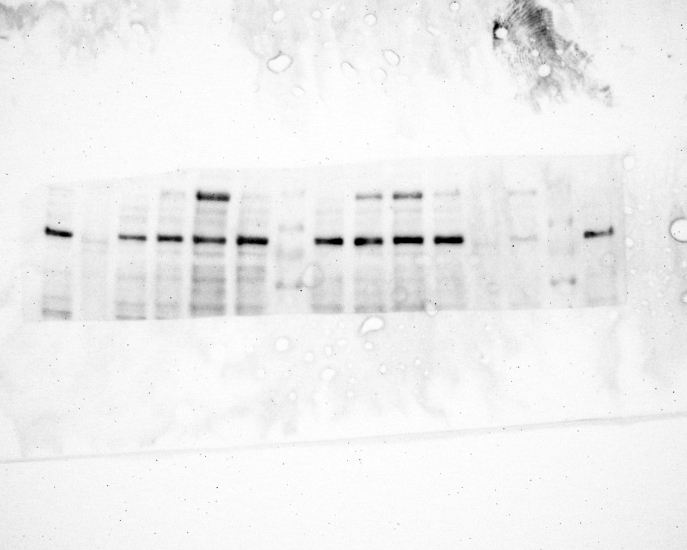

Supplement: Figure 3—figure supplement 3—source data 1. [file elife-85690-fig3-figsupp3-data1.zip › Figure 3- figure supplement 3- source data 1/anti-DNAK-oct13_2022_3(Chemiluminescence).raw16.tif]

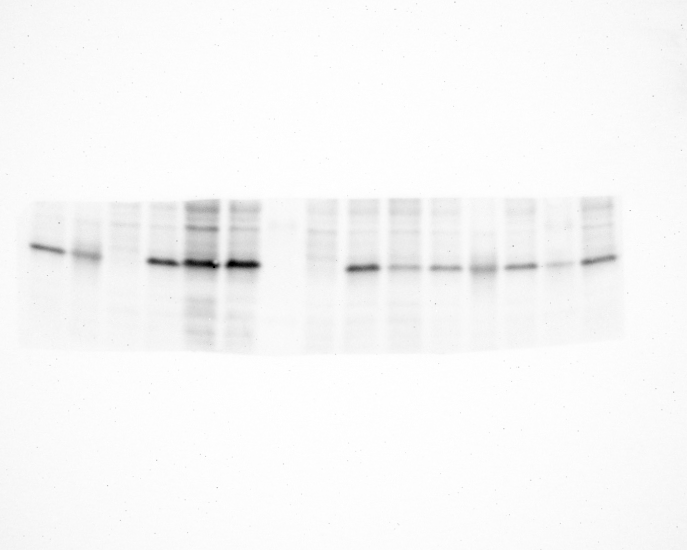

Supplement: Figure 3—figure supplement 3—source data 1. [file elife-85690-fig3-figsupp3-data1.zip › Figure 3- figure supplement 3- source data 1/anti-histag_oct13-2022_1(Chemiluminescence).raw16.tif]

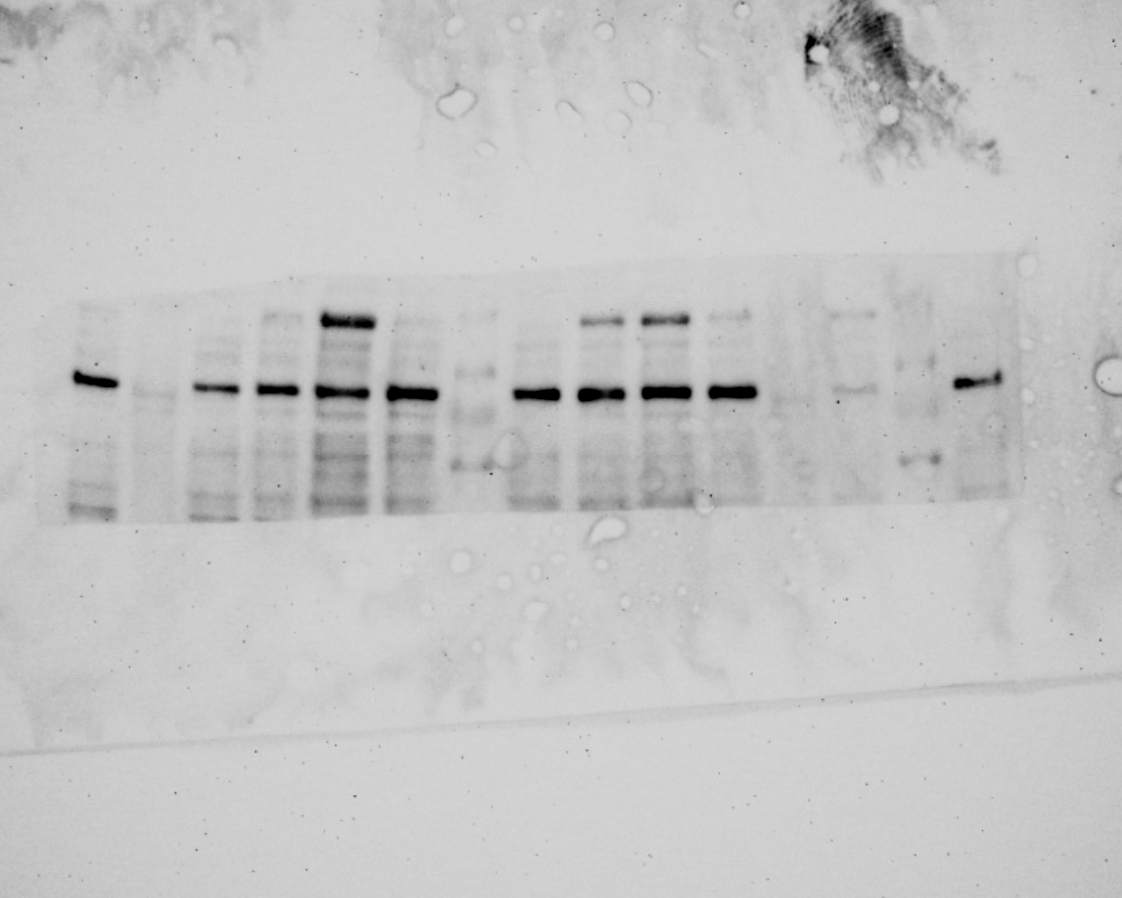

Supplement: Figure 3—figure supplement 3—source data 1. [file elife-85690-fig3-figsupp3-data1.zip › Figure 3- figure supplement 3- source data 1/anti-DNAK-oct13_2022_3(Chemiluminescence).tif]

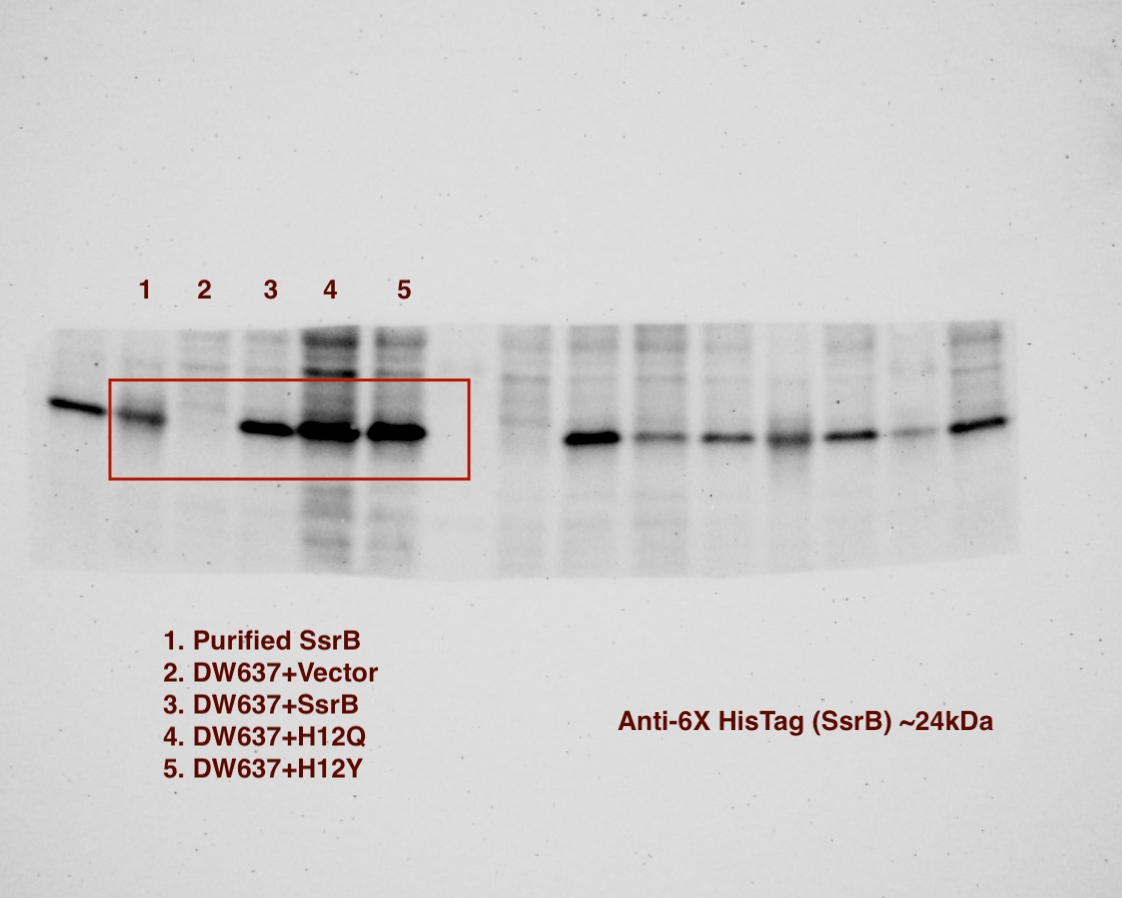

Supplement: Figure 3—figure supplement 3—source data 1. [file elife-85690-fig3-figsupp3-data1.zip › Figure 3- figure supplement 3- source data 1/anti-histag_oct13-2022_1(Chemiluminescence).jpg]

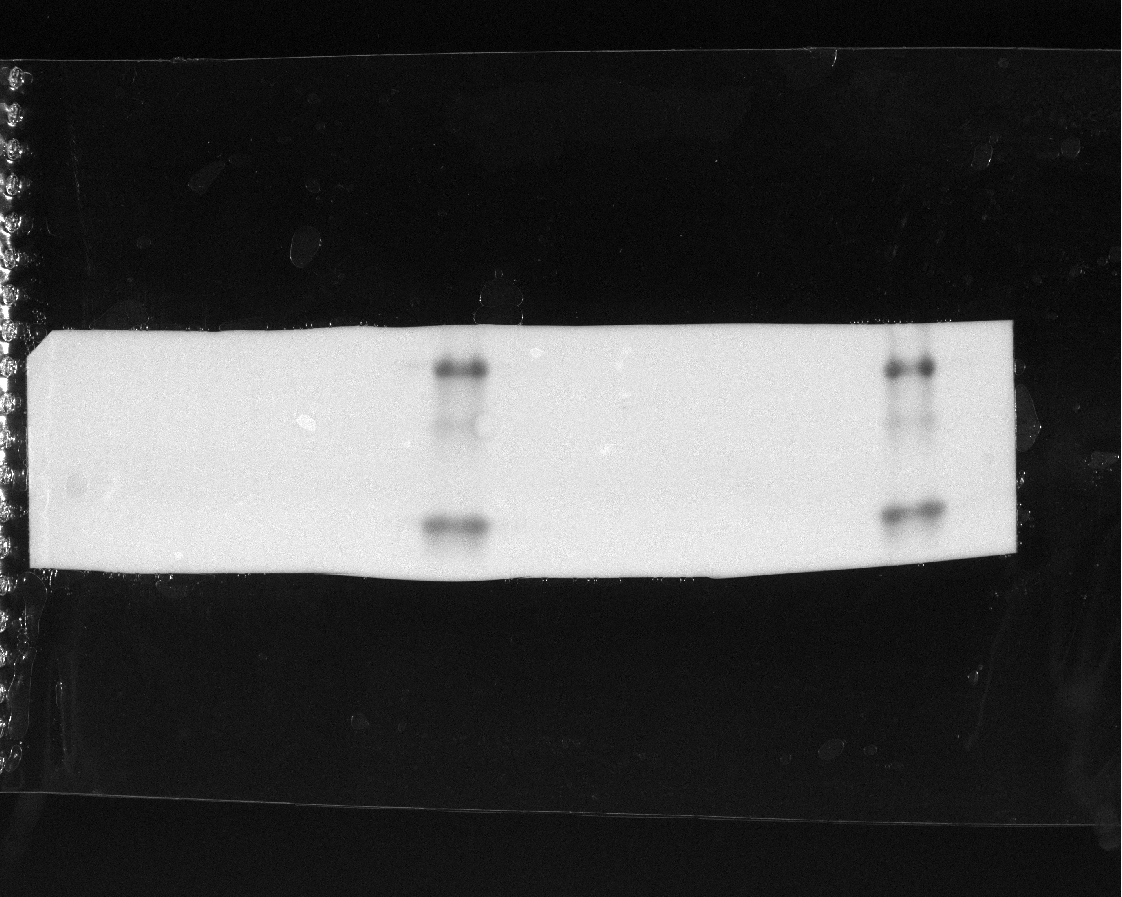

Supplement: Figure 3—figure supplement 3—source data 1. [file elife-85690-fig3-figsupp3-data1.zip › Figure 3- figure supplement 3- source data 1/anti-histag_oct13-2022_4(Colorimetric).tif.tif]

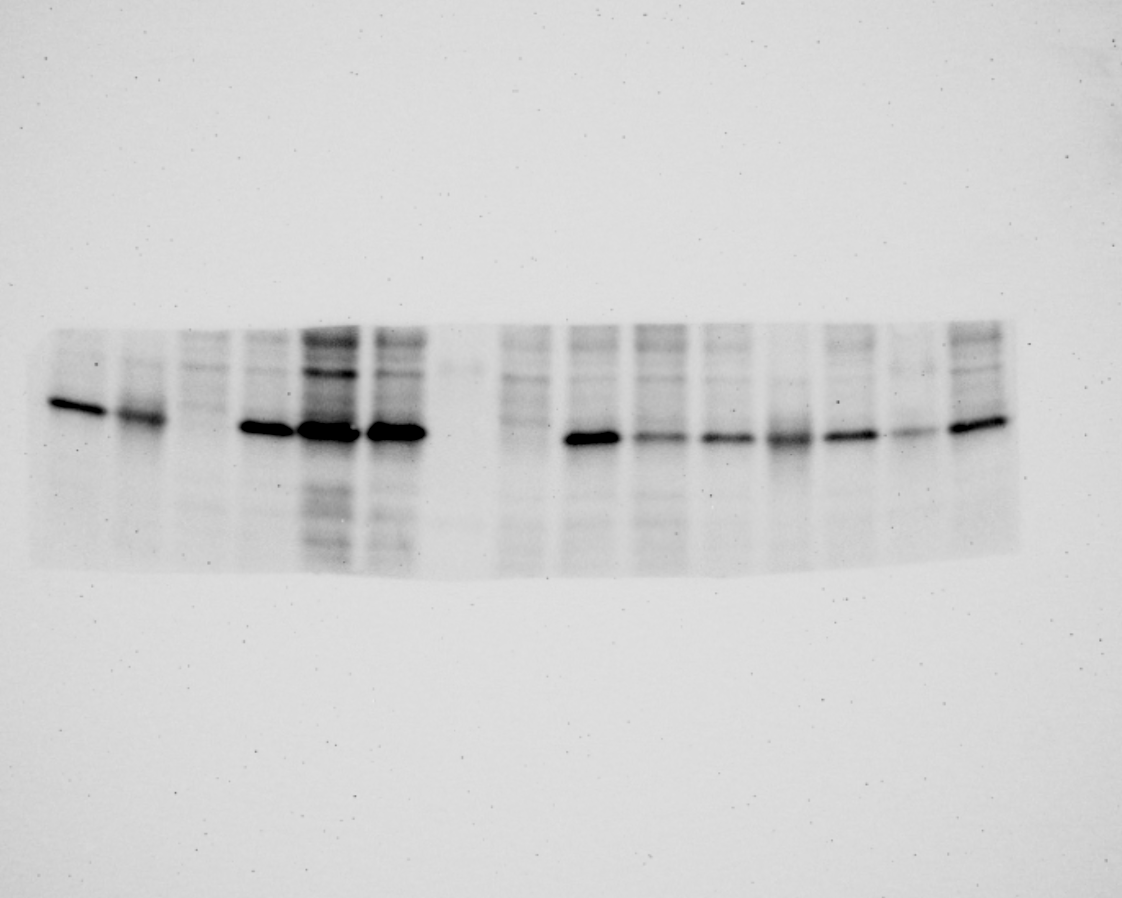

Supplement: Figure 3—figure supplement 3—source data 1. [file elife-85690-fig3-figsupp3-data1.zip › Figure 3- figure supplement 3- source data 1/anti-histag_oct13-2022_1(Chemiluminescence).tif]

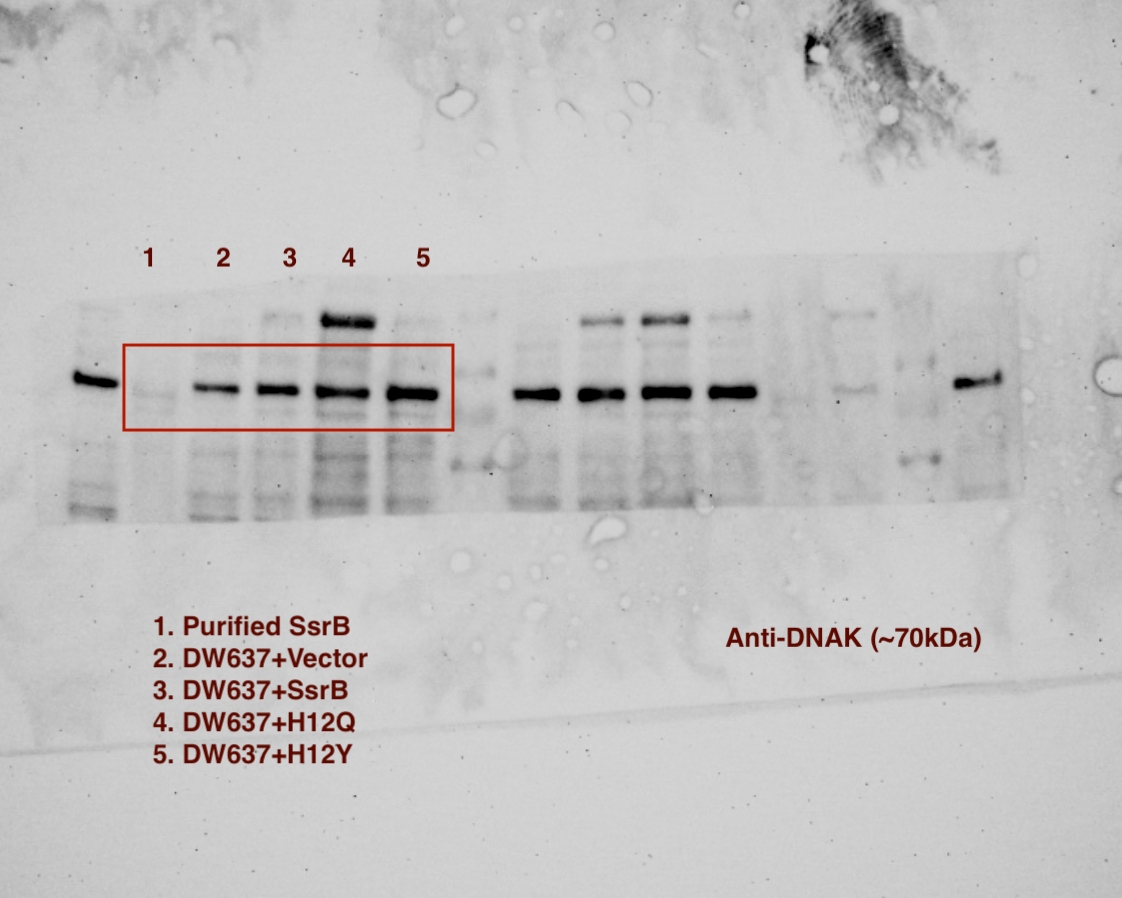

Supplement: Figure 3—figure supplement 3—source data 1. [file elife-85690-fig3-figsupp3-data1.zip › Figure 3- figure supplement 3- source data 1/anti-DNAK-oct13_2022_3(Chemiluminescence).jpg]
